# Novel relatives of Mecsek Mountains mammarenavirus (family *Arenaviridae*) in hedgehogs living in different sampling areas in Hungary

**DOI:** 10.1038/s41598-025-87108-2

**Published:** 2025-01-23

**Authors:** Károly Takáts, Péter Pankovics, Benigna Balázs, Ákos Boros, Róbert Mátics, Gábor Reuter

**Affiliations:** 1 Department of Medical Microbiology and Immunology, Medical School, University of Pécs, Szigeti út 12, Pécs, 7624 Hungary; 2 Hungarian Nature Research Society, Ajka, Hungary; 3 Department of Behavioural Sciences, Medical School, University of Pécs, Pécs, Hungary

**Keywords:** Arenavirus, Mammarenavirus, *Arenaviridae*, Mecsek Mountains virus, Pannonia mammarenavirus, Hedgehog, Europe, Microbiology, Molecular biology

## Abstract

Mammarenaviruses (genus *Mammarenavirus*, family *Arenaviridae*) are rodent-borne zoonotic viruses consisting of 52 viral species, including ten that are pathogenic to humans. Currently, only two endemic mammarenavirus species are known in Europe: the human pathogenic *Mammarenavirus choriomeningitidis* (LCMV) and the recently discovered hedgehog-origin *Mammarenavirus mecsekense* (MEMV). In this study, 59 faecal specimens from Northern white-breasted hedgehogs (*Erinaceus roumanicus*) from different geographic regions in Hungary were investigated for mammarenavirus presence and complete genome characterization using newly designed screening primers by RT-semi-nested PCR and sequencing methods. Five (8.5%) of the 59 samples tested positive for mammarenavirus RNA (ER8, ER15, ER27, ER33, and ER39, GenBank accession numbers PQ441959-PQ441968). The L- and S-segments of these strains showed 66–93% and 73–92% nt identity to the closest known mammarenavirus, MEMV, respectively. The NP protein exhibited 86–97% aa sequence identity compared to the corresponding protein of MEMV. Notably, the S-segment intergenic region (S-IGR) of strains ER8, ER15, ER27 and ER33 exceeded the average nt length among known mammarenaviruses and contained two, highly similar stem-loop structures with conserved self-complementary nucleotide motifs. Based on the sequence- and phylogenetic analysis these strains (ER8, ER15, ER27 and ER33) potentially represent a novel mammarenavirus species, tentatively named Pannonia mammarenavirus (PANV).

**Supplementary Information:**

The online version contains supplementary material available at 10.1038/s41598-025-87108-2.

## Introduction

Arenaviruses (family *Arenaviridae*) are enveloped viruses with segmented, ambisense linear RNA genomes comprising approximately 10.5 kb in length. Currently, the family is divided into five genera: *Antennavirus*, *Hartmanivirus*, *Innmovirus*, *Mammarenavirus* and *Reptarenavirus* comprising 69 officially classified species^1^ (https://ictv.global/report/chapter/arenaviridae/arenaviridae). The arenavirus genome consists of two (L and S in genera *Mammarenavirus*,* Hartmanivirus and Reptarenavirus*) or three (L, M and S in genera *Antennavirus* and *Innmovirus*) genomic segments encoding three (in genera *Hartmanivirus* and *Innomovirus*), or four (in genera *Mammarenavirus*,* Reptarenavirus* and *Antennavirus*) viral proteins in non-overlapping open reading frames (ORF) of opposite polarities (called ambisense coding arrangement). The ORFs are separated by a non-coding intergenic region (IGR) forming stem-loop (hairpin) structures. These structures are critical for transcription termination, as well as for virion assembly and release of new viral particles^2^. The S-segment encodes the nucleoprotein (NP) and the glycoprotein precursor protein (GP) except in genera *Antennavirus* and *Innmovirus*, where these viral proteins encoded on two separate segments (S and M). The L-segment encodes the L protein (RNA-dependent RNA polymerase) in all known arenaviruses and the Z protein in genera *Mammarenavirus* and *Reptarenavirus*^1^. The GP is enzymatically cleaved into three functional subunits: stable signal peptide (SSP), glycoprotein 1 (GP1) and glycoprotein 2 (GP2)^3,4^.

The geographic distribution of arenaviruses are in close relation with the distribution of their specific natural hosts. While rodents are traditionally seen as primary hosts for arenaviruses, recent studies have expanded this host spectrum to include other animals. According the current knowledge, members of a given arenavirus genus only occur in specific natural hosts. For instance, viruses in the genera *Hartmanivirus* and *Reptarenavirus* infect snakes^5^ while the genus *Antennavirus* infect fish^6^. The natural reservoir for innmoviruses remains unknown^7^. Small rodents (order *Rodentia*) belonging to the family *Muridae* (Old World), and family *Cricetidae* (New World) are the main reservoir of arenaviruses in the genus *Mammarenavirus*^8^. However, mammarenaviruses were also reported from bats (order *Chiroptera*)^9,10^, ticks (order *Ixodida*)^11^, shrews (order *Eulipotyphla*)^12^, three-toed jerboas (order *Rodentia*)^13^, and recently pikas (order *Lagomorpha*)^8^, and hedgehogs (order *Eulipotyphla*)^14^.

Mammarenavirus infections are generally asymptomatic in their natural rodent hosts. However, in humans, some mammarenaviruses are known pathogens, such as Lassa virus (LASV) and Lujo virus in Africa and six mammarenavirus species of American origin that can cause severe and frequently fatal diseases with hemorrhagic manifestations^15^. Lymphocytic choriomeningitis virus (LCMV, *Mammarenavirus choriomeningitidis*), a typical mammalian arenavirus with worldwide distribution, can cause disease in the central nervous system (meningitis and meningoencephalitis) and poses a serious threat to immunocompromised individuals and the fetus in pregnancy^16^.

The Mecsek Mountains mammarenavirus (MEMV) is the second known arenavirus endemic in Europe beside lymphocytic choriomeningitis virus (LCMV)^14^. This mammarenavirus was discovered in faecal specimens collected from Northern white-breasted hedgehogs (*Erinaceus roumanicus*) in Hungary in 2023^14^. MEMV represents a novel mammarenavirus species called *Mammarenavirus mecsekense*^1,17^.

Given that the novel MEMV mammarenavirus species was identified from an animal previously not known as a host species, we extended our study. Further faecal specimens collected from hedgehogs living in different sampling areas in Hungary were investigated for arenaviruses. In this study, we report the detection and complete genome characterization of additional, novel mammarenaviruses from hedgehogs.

## Results

### Novel mammarenaviruses from hedgehogs in Hungary

**Fig. 1:**
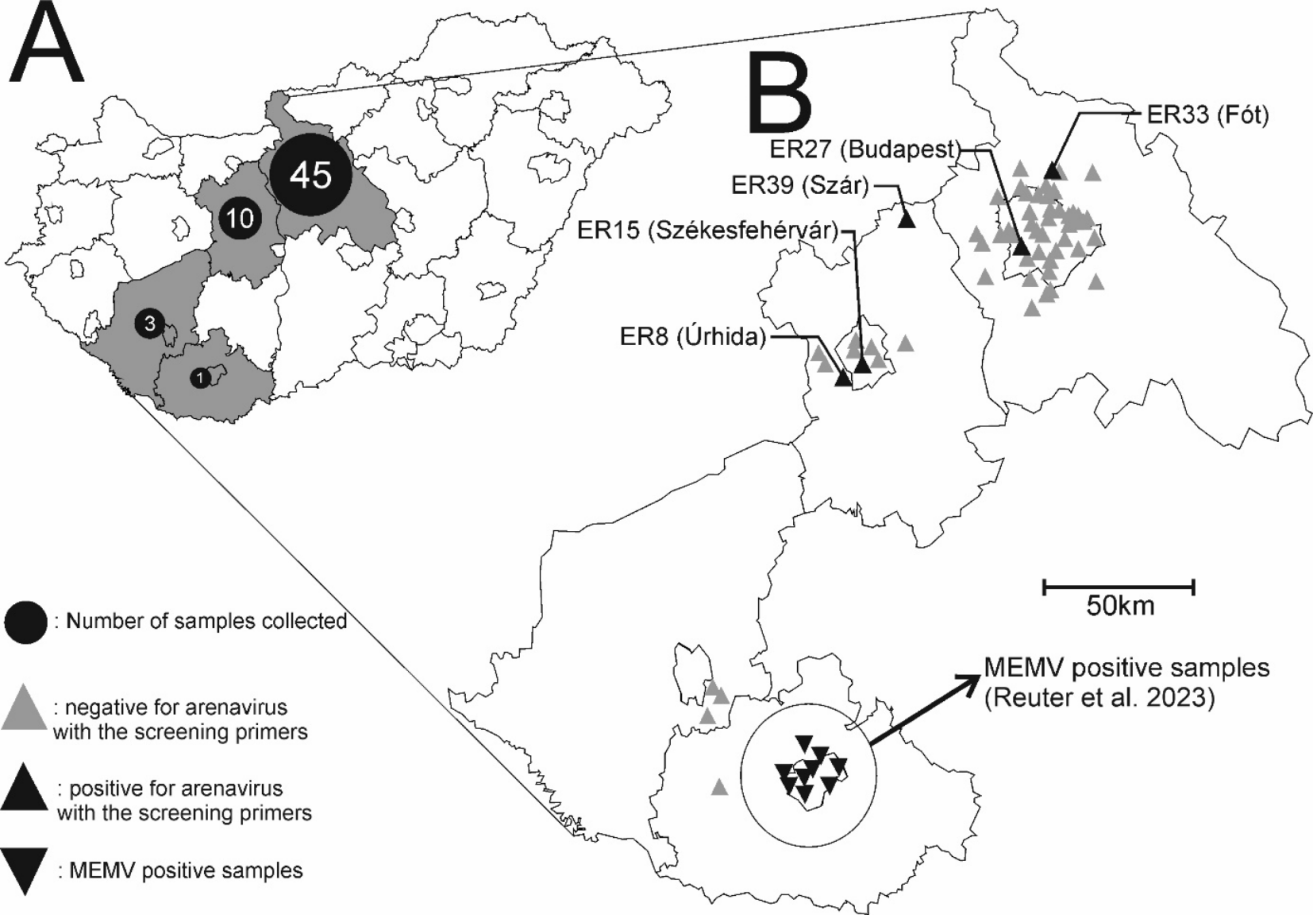
(**A**) Geographic distribution of the faecal specimens (N=∑59) collected from hedgehogs (Erinaceus roumanicus) in this study, from 4 counties (Baranya *N* = 1, Fejér *N* = 10, Pest *N* = 45, and Somogy *N* = 3) in Hungary. (**B**) Triangle symbols in the magnified image represent mammarenavirus negative (, *N* = 54) and positive (▲, *N* = 5) samples by RT-nested-PCR and Sanger-sequencing methods. The sample ID and the name of the sampling settlement (in brackets) are indicated in positive samples. The inverted triangle (▼) indicates the collection location of the Mecsek Mountains virus (MEMV)-positive samples (*N* = 9) from the previous study^14^. The blank map of Hungary was downloaded from https://mapsvg.com/maps/hungary and modified by the authors using CorelDRAW 2021. version 23.1.0.389. The program is licensed through the University of Pécs.

A total of 59 faecal samples were collected from Northern white-breasted hedgehogs (*Erinaceus roumanicus* Barrett-Hamilton, 1900) from different geographical locations (Baranya, Fejér, Pest including the capital Budapest, and Somogy counties) in Hungary (Table S1; Fig. 1). Using the newly designed mammarenavirus screening primers for the L-segment, 5 (8.5%) of the 59 samples were positive for mammarenavirus by RT-semi-nested PCR and Sanger-sequencing methods in two geographic areas (Fig. 1). The mammarenavirus-positive samples were collected from animals in settlements Úrhida (ER8), Székesfehérvár (ER15), the capital Budapest (ER27), Fót (ER33), and Szár (ER39) (Fig. 1).

The complete mammarenaviral coding genome sequences (L- and S-segments) were determined in all the five study strains (ER8/2023/HUN, PQ441959-PQ441960; ER15/2023/HUN, PQ441961-PQ441962; ER27/2023/HUN, PQ441963-PQ441964; ER33/2023/HUN, PQ441965-PQ441966 and ER39/2023/HUN, PQ441967-PQ441968). The detailed nt and aa sequence lengths and sequence identities in percentage of hedgehog mammarenaviruses from Hungary compared with the prototype MEMV (OP191655, OP191656)^14^ are shown in Table 1 (Table 1). The L- and S-segments were variable in length between 7,389 and 7,464 nt and between 3,536 and 3,684 nt, respectively, representing the longest mammarenavirus genomes published so far (e.g. ER27/2023/HUN has 11,142 nt long total genome length). Except for the sequence length of the Z protein, all the other genome parts and viral proteins are different in length among the study strains (Table 1). All the known conserved mammarenavirus aa sequence motifs (e.g. type-II RNA endonuclease motif in RdRp; conserved functional sites in Z protein; SSP/GP1 and GP1/GP2 cleavage sites in GP)^4,18–21^ are present in the viral proteins of the study strains. Same as in MEMV^14^, there is a 37-aa-long extension in the N-terminal part (aa positions 5 to 41) of the NP protein with unknown function in all the study strains. In addition, there is a lysine (K) and glutamic acid (E) rich part in the highly variable region of NP upstream at aa 383 in the study strains with repeated aa motif KE (e.g. K_383_EKDTKEKEKEKEK_396_ in strain ER15). Based on the seven terminal GP1 aa residues (V_251_ITRRLQ↓), the study strains are rather be an α-dystroglycan (α-DG) than transferrin receptor protein 1 (TfR1) cellular receptor-tropic arenaviruses similar to Lassa virus and LCMV^3,4^.

**Table 1:** Nucleotide (nt) and amino acid (aa) sequence lengths and sequence comparison of ER8/2023/HUN, ER15/2023/HUN, ER27/2023/HUN, ER33/2023/HUN and ER39/2023/HUN mammarenavirus L- and S-segments, compared to the corresponding regions of the prototype MEMV (OP191655, OP191656).

L-segment	MEMV (OP191655)	ER8 (PQ441959)	ER15 (PQ441961)	ER27 (PQ441963)	ER33 (PQ441965)	ER39 (PQ441967)
Complete genome nt (nt% to MEMV)	7,393 (100%)	7,407 (68%)	7,403 (68%)	7,464 (67%)	7,460 (66%)	7,389 (93%)
3’UTR nt	78	132	131	188	188	79
RdRp nt/aa (nt%/aa% to MEMV)	6,726/2,241 (100%/100%)	6,690/2,229 (70%/70%)	6,690/2,229 (70%/71%)	6,696/2,231 (69%/71%)	6,693/2,230 (69%/71%)	6,720/2,239 (94%/96%)
IGR nt	216	233	229	224	228	216
Z-protein nt/aa (nt%/aa% to MEMV)	276/91 (100%/ 100%)	276/91 (74%/82%)	276/91 (73%/82%)	276/91 (73%/80%)	276/91 (71%/80%)	276/91 (97%/97%)
5’UTR nt	97	76	77	80	75	98

S-segment	MEMV (OP191656)	ER8 (PQ441960)	ER15 (PQ441962)	ER27 (PQ441964)	ER33 (PQ441966)	ER39 (PQ441968)
Complete genome length nt (nt% to MEMV)	3,536 (100%)	3,684 (73%)	3,682 (73%)	3,678 (73%)	3,679 (73%)	3,530 (92%)
3’UTR nt	81	81	81	80	81	81
NP nt/aa (nt%/aa% to MEMV)	1,824/607 (100%/100%)	1,833/610 (78%/87%)	1,833/610 (78%/87%)	1827/608 (78%/87%)	1827/608 (77%/86%)	1,824 /607 (94%/97%)
IGR nt	96	235	233	236	236	90
GP nt/aa (nt%/aa% to MEMV)	1,464/487 (100%)	1,464/487 (76%/85%)	1,464/487 (77%/84%)	1,464/487 (78%/84%)	1,464/487 (77%/82%)	1,464/487 (93%/96%)
5’UTR nt	71	71	71	71	71	71

UTR, untranslated region; RdRp, RNA-dependent RNA polymerase; IGR, intergenic region; NP, nucleoprotein; GP, glycoprotein.

The L-segments of the study strains had 66–93% nt identities to the closest mammarenavirus, MEMV (OP191655), with the lowest identity (66%) to ER33/2023/HUN (Table 1). The RdRp proteins of the study strains have 70–96% aa sequence identity to the corresponding protein of MEMV with the lowest identity (70%) to ER8/2023/HUN (Table 1). The Z protein of the study strains have 80–97% aa sequence identity to the the corresponding protein of MEMV (Table 1).

The S-segments of the study strains had 73–92% nt identities to the closest mammarenavirus, MEMV (OP191656), with the lowest identity (73%) to ER8/2023/HUN, ER15/2023/HUN, ER27/2023/HUN and ER33/2023/HUN (Table 1). The NP protein of the study strains have 86–97% aa sequence identity to the corresponding protein of MEMV with the lowest identity (86%) to ER33/2023/HUN (Table 1). The GP proteins of the study strains have 82–96% aa sequence identity to the corresponding protein of MEMV with the lowest identity (82%) to ER33/2023/HUN (Table 1).

**Fig. 2:**
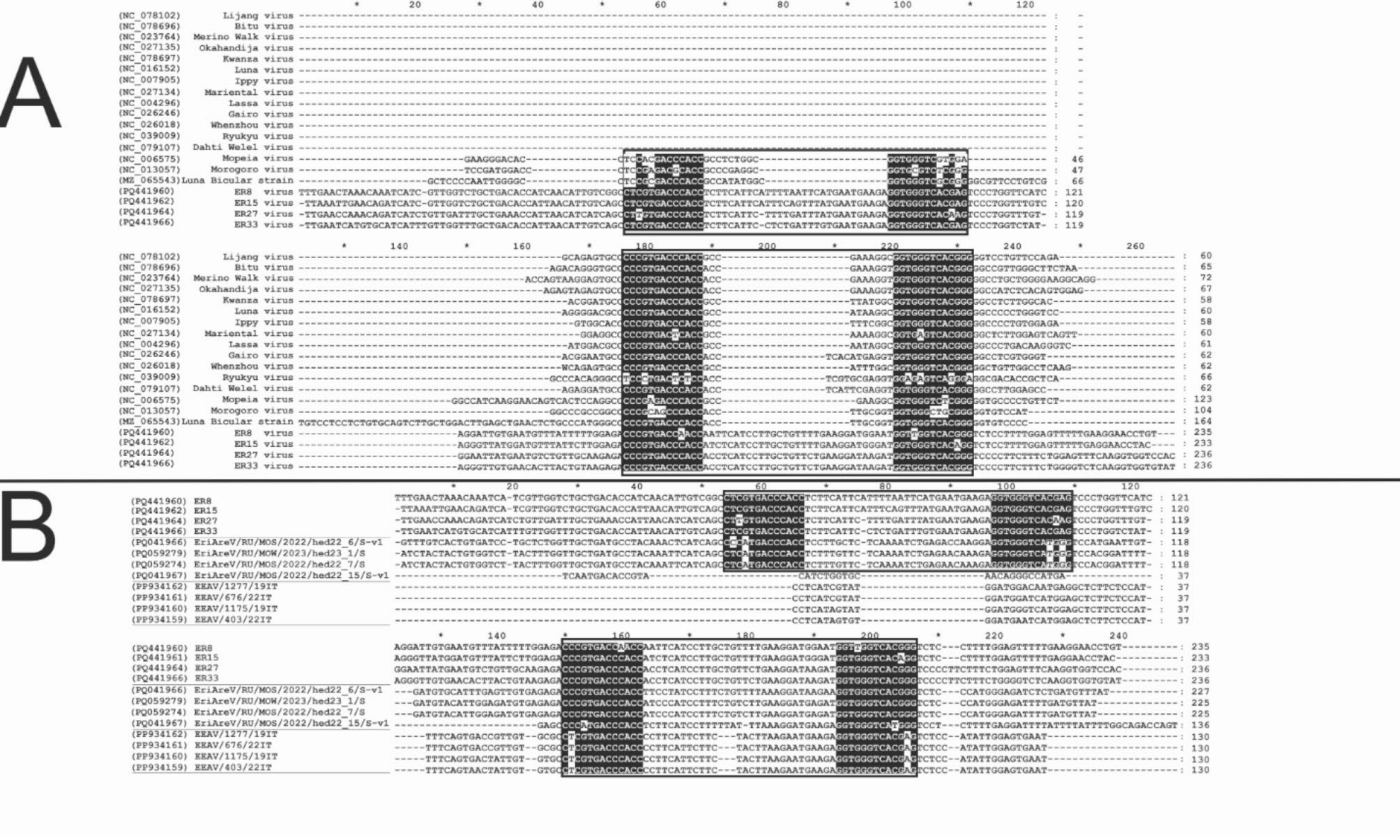
Multiple nt sequence alignment of the mammarenavirus S-segment intergenic regions (S-IGR). (**A**) S-IGR alignment of ER8/2023/HUN, ER15/2023/HUN, ER27/2023/HUN and ER33/2023/HUN study strains with selected Old World mammarenavirus S-IGRs. The S-IGR sequences of ER8/2023/HUN, ER15/2023/HUN, ER27/2023/HUN and ER33/2023/HUN (and the Mopeia, Morogoro and Luna Bicuar mammarenaviruses) contain a putative highly conserved, self-complementary, twin nt motifs (the more general formula of this 13-nt-long sequence motifs are YYYVHRRCBMWCC and GGWKVGYYDBRRR in the selected S-IGRs) (black background) which form stem-loop (SL) structures (black frame, Fig. 3). (**B**) S-IGR alignment of ER8/2023/HUN, ER15/2023/HUN, ER27/2023/HUN and ER33/2023/HUN with hedgehog mammarenaviruses from Italy and Russia. The double self-complementary nt motifs (black background) and stem-loops (black frame) are also present in three hedgehog mammarenavirus S-IGR sequences from Russia.

**Fig. 3:**
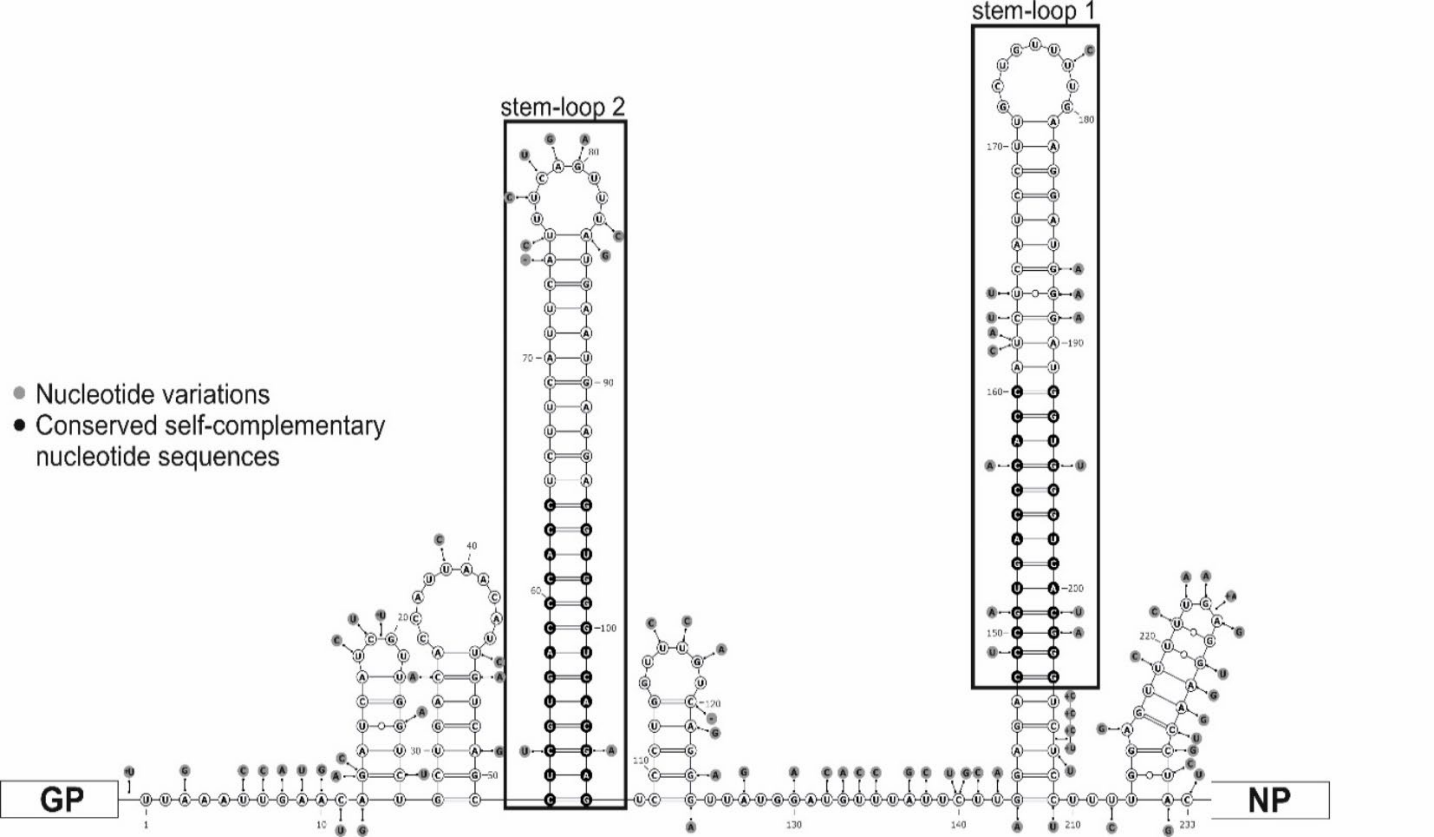
Predicted secondary RNA structure of the S-segment intergenic regions (S-IGR) based on the superimposed nucleotide sequences of ER8/2023/HUN, ER15/2023/HUN, ER27/2023/HUN and ER33/2023/HUN. The most characteristic structure is the double stem-loop structure (stem-loop 1 and stem-loop 2 in black frame) with the highly conserved self-complementary nt motifs (Fig. 2) indicated by black circles. Presumably, the stem-loop 1 structure is the common, primary stem-loop in Old Word mammarenaviruses. Positions of nucleotide sequence variations in the four strains are shown by grey circles. GP: glycoprotein-coding region; NP: nucleoprotein-coding region.

The nt length of the 3’ and 5’ non-coding regions of the L-segment of the study strains, especially in ER27/2023/HUN and ER33/2023/HUN, exceeds the average nt length among mammarenaviruses (e.g. the 3’ non-coding region is 188 nt in ER27/2023/HUN and ER33/2023/HUN) (Table 1). In addition, the nt length of the IGR region of the S-segment (S-IGR) is significantly longer (233–236 nt in length versus 96 nt in MEMV) in ER8/2023/HUN, ER15/2023/HUN, ER27/2023/HUN and ER33/2023/HUN than in the known mammarenaviruses (Table 1). Multiple sequence alignment and the predicted RNA secondary structure analysis of this non-coding region by RNAfold (ViennaRNA Web Services) revealed that the S-IGR region of ER8/2023/HUN, ER15/2023/HUN, ER27/2023/HUN and ER33/2023/HUN contains two, highly similar stem-loop (SL1 and SL2) structures with conserved self-complementary nt motifs CCYGUGACCMACC and GGUKGGUCACRGG (the more general formula of this complementary mammarenavirus sequence motifs are YYYVHRR**C**BMW**CC** and **GG**WKV**G**YYDBRRR where bold letters indicate highly conserved nucleotides) (Figs. 2A and 3).

### Additional novel mammarenaviruses in hedgehogs from other parts of Eurasia

**Fig. 4:**
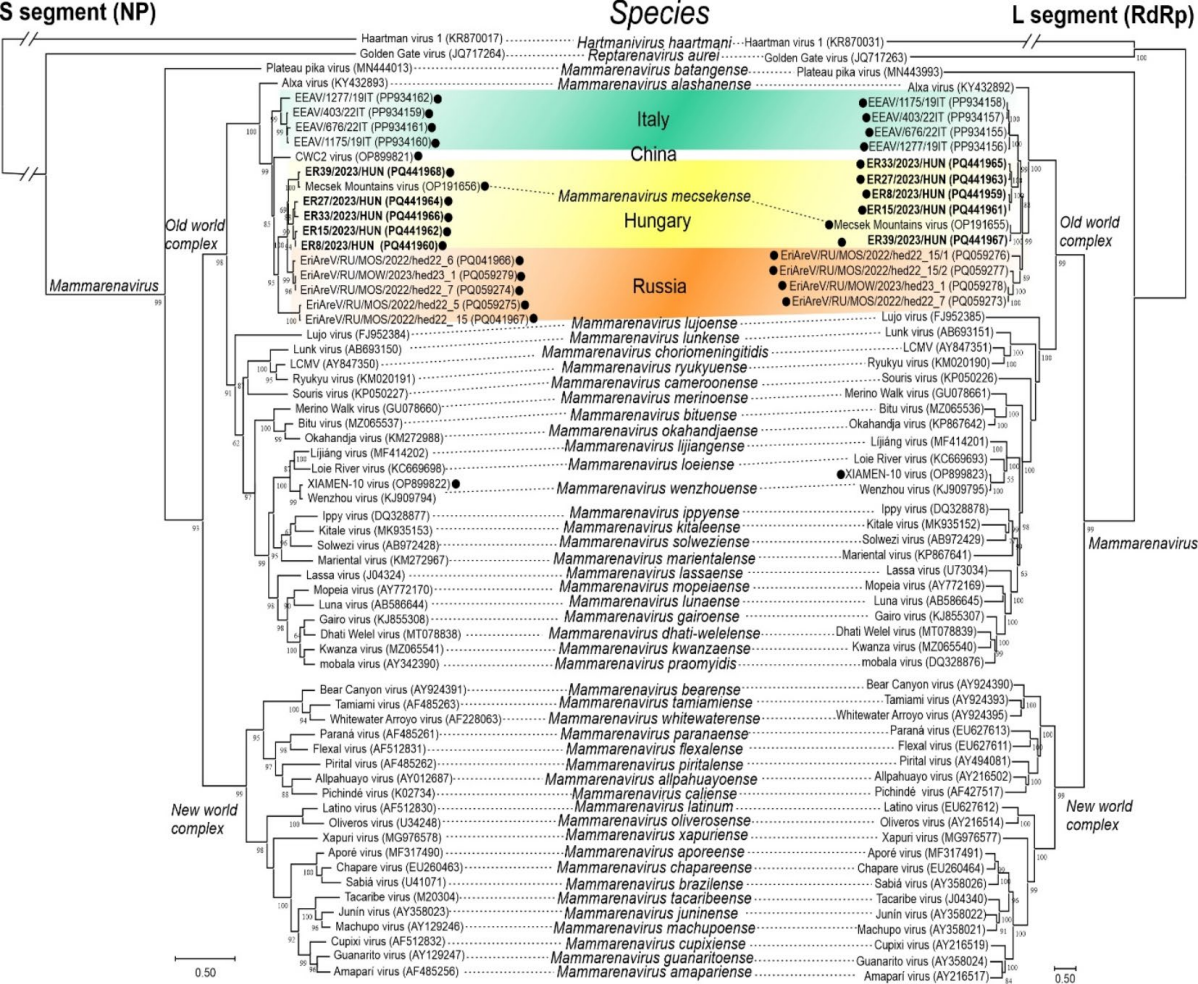
Maximum likelihood phylogenetic trees were generated from webPRANK alignments^25^ of the S-segment/nucleoprotein (NP) (left) and L-segment/RNA-dependent RNA-polymerase, RdRp (right) amino acid (aa) sequences of mammarenaviruses using the best-fit model of protein evolution (LG + G) selected previously^26^. The trees with 1,000 ultrafast bootstrap replicates were produced using the IQ-Tree web server^27^ and visualized by MEGAX^28^ and Corel Draw Standard 2020. Study sequences are marked in **bold**. Dark circles indicate the mammarenaviruses described from hedgehogs. Countries of origins of various hedgehog mammarenaviruses were indicated on the trees and marked with different background colors. Hedgehog mammarenavirus XIAMEN-10 (OP899822) from China belongs to the species *Mammarenavirus wenzhouense*. LCMV: lymphocytic choriomeningitis virus.

**Fig. 5:**
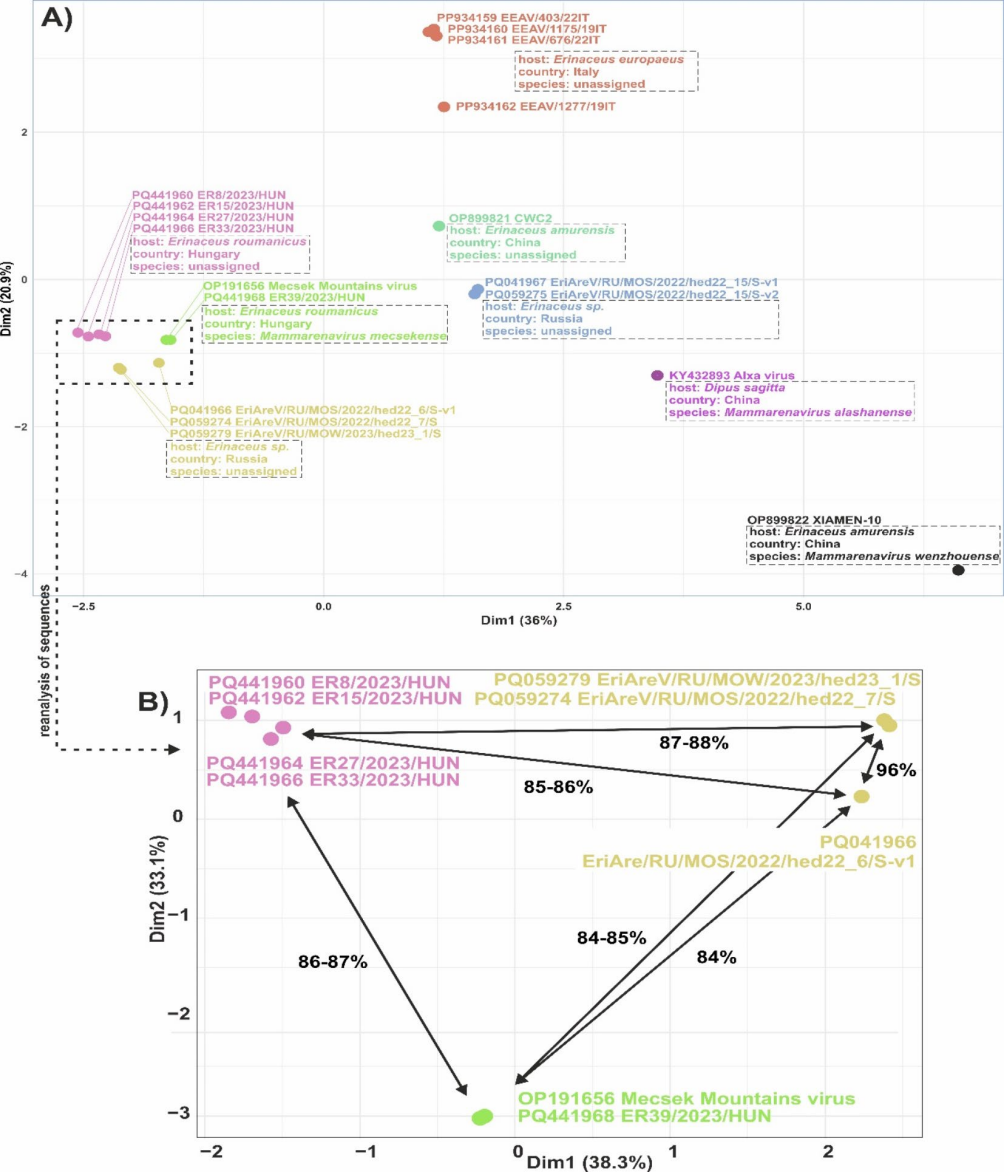
Hierarchical clustering of the complete NP protein of novel and representative mammarenavirus sequences. Panel (**A**) depicts the groups of mammarenavirus species, including *Mammarenavirus mecsekense* (MEMV), *Mammarenavirus alashanense*, and *Mammarenavirus wenzhouense*, as officially approved by the International Committee on Taxonomy of Viruses (ICTV) and it shows mammarenavirus sequences currently awaiting classification, such as EriAreV/RU/MOS/2022-hed22_15/S-v1, EriAreV/RU/MOS/2022/hed22_15/S-v2, CWC2, EEAV/1277/19IT, EEAV/676/22IT, EEAV/1175/19IT, EEAV/403/22IT, ER8, ER15, ER27, ER33, ER39, EriAreV/RU/MOS/2022/hed22_6/S-v1, EriAreV/RU/MOS/2022/hed22_7/S, and EriAreV/RU/MOW/2023/hed23_1/S, grouped by hierarchical clustering. Panel A also includes information on the country of origin, the host species (where available) for each sequence. All potential novel and officially approved mammarenavirus species from hedgehogs are represented by a distinct colour. Panel (**B**) shows the re-analysis of selected sequences (MEMV, ER8/2023/HUN, ER15/2023/HUN, ER27/2023/HUN, ER33/2023/HUN, ER39/2023/HUN, EriAreV/RU/MOW/2023/hed23_1/S, EriAreV/RU/MOS/2022/hed22_7/S, and EriAre/RU/MOS/2022/hed22_6/S-v1) using the same R script. In addition to visualizing the hierarchical clustering, this panel includes amino acid identity values for the analysed sequences and their closest genetic relatives, as determined in this study. The hierarchical cluster analysis was performed the “cluster_analysis” script written in RStudio, version (RStudio 2024.09.0 + 375) using the ‘ape’, ‘cluster’, ‘cowplot’, ‘factoextra’, ‘ggrepel’, ‘gridExtra’, ‘msa’, ‘plotly’, ‘randomcoloR’ and ‘seqinr’ R packages. The plots generated from the analysis in RStudio and were post-edited using the CorelDRAW software. Briefly, as the first step of hierarchical clustering, the amino acid sequences were loaded, followed by performing multiple sequence alignment using the ClustalW method. The amino acid distances between the sequences were calculated, the data were scaled, and the hierarchical clustering was performed using the complete linkage method. To determine the optimal groups, the ‘silhouette,’ ‘Elbow/WSS (Within-Cluster Sum of Squares)’ and GapStatistic methods from the factoextra package’s fviz_nbclust function were applied, along with consideration of the genetic distances between the sequences. The Dim1 and Dim2% values displayed along the axes represent the proportion of total variance explained by each axis. A higher percentage on an axis indicates that the corresponding dimension captures a greater portion of the variability in the data, while lower percentages suggest that less variability is captured^29,30^. The “cluster_analysis” script is available upon request.

In the sequence (Tables 2 and 3) and phylogenetic (Fig. 4) analyses, unpublished hedgehog mammarenavirus L- and S-segment sequences deposited in July 2024 in GenBank were also used. This includes novel mammarenavirus sequences identified in liver specimens from European hedgehogs (*Erinaceus europeus*) from Italy (PP934155-PP934162, Di Martino et al., unpublished data) and in oral and anal swab samples from undetermined hedgehog species from Russia (PQ059273-PQ059279, PQ041966-PQ041967, Lukina-Gronskaya et al., unpublished data), respectively. In addition, mammarenavirus sequences (OP899820-OP899823, Zhu et al., unpublished data) were also available from hedgehogs (*Erinaceus amurensis*) from China, in June 2023. The L- and S-segments of the hedgehog mammarenavirus strains from Hungary have 60–68% and 50–78% nt identities to the corresponding sequences from Russia, Italy and China (Table 2). The NPs have 53–88% aa identities to the corresponding sequences from Russia, Italy and China (Table 3). Phylogenetic analysis based on the full-length aa sequences of the RdRp (L-segment) and NP (S-segment) showed that (except XIAMEN-10 OP899822-OP899823 from China which belongs to *Mammarenavirus wenzhouense*) all other hedgehog mammarenavirus sequences from Hungary, Italy, Russia and China related to a clade that includes the Mecsek Mountains virus (species *Mammarenavirus mecsekense*) and Alxa virus (species *Mammarenavirus alashanense*) (Fig. 4). These mammarenavirus sequences are clustered according to their geographic origin and (based on sequence and phylogenetic analyses) could potentially be separated into up to 5 novel mammarenavirus species (Figs. 4 and 5, Table S2). By sequence analysis, three hedgehog mammarenavirus S-IGR sequences (PQ041966, PQ059274 and PQ059279) from Russia also contain the twin stem-loop structures with the conserved self-complementary nt motifs (Fig. 2B).

**Table 2:** Nucleotide sequence comparison of hedgehog mammarenavirus L- and S-segments with complete or partial coding regions from Russia, Italy and China compared to the sequences (MEMV, ER8, ER15, ER27, ER33 and ER39) from Hungary.

Hedgehog mammarenavirus L-segment nucleotide sequence(s) from	GenBank accession numbers	nt identity in % to					
		MEMV (OP191655)	ER8 (PQ441959)	ER15 (PQ441961)	ER27 (PQ441963)	ER33 (PQ441965)	ER39 (PQ441967)
Russia	PQ059273	66%	66%	66%	65%	66%	66%
	PQ059276	66%	67%	66%	66%	67%	66%
	PQ059277	66%	67%	67%	66%	66%	66%
	PQ059278	67%	66%	66%	66%	66%	66%
Italy	PP934155	67%	68%	68%	67%	67%	67%
	PP934156	67%	68%	68%	67%	67%	67%
	PP934157	66%	67%	68%	67%	67%	66%
	PP934158	66%	68%	68%	67%	67%	66%
China	OP899820 (partial)	60%	61%	61%	60%	60%	60%

Hedgehog mammarenavirus S-segment nucleotide sequence(s) from	GenBank accession numbers	nt identity in % to					
		MEMV (OP191656)	ER8 (PQ441960)	ER15 (PQ441962)	ER27 (PQ441964)	ER33 (PQ441966)	ER39 (PQ441968)
Russia	PQ041966	72%	78%	78%	78%	78%	74%
	PQ041967 (partial)	69%	67%	67%	67%	67%	70%
	PQ059274	71%	78%	77%	77%	77%	73%
	PQ059275 (partial)	67%	66%	65%	65%	64%	68%
	PQ059279	69%	75%	75%	76%	76%	70%
Italy	PP934159	71%	71%	71%	70%	71%	72%
	PP934160	71%	70%	71%	70%	71%	72%
	PP934161	71%	70%	70%	70%	71%	72%
	PP934162	71%	70%	70%	71%	70%	72%
China	OP899821	65%	64%	65%	65%	65%	66%
	OP899822*	52%	50%	50%	50%	50%	52%

*Hedgehog mammarenavirus XIAMEN-10 (OP899822) from China belongs to the species *Mammarenavirus wenzhouense*.

**Table 3:** Amino acid (aa) sequence comparison of the hedgehog mammarenavirus nucleoprotein (NP) sequences from Russia, Italy and China compared to sequences (MEMV, ER8, ER15, ER27, ER33 and ER39) from Hungary.

NP aa sequence of hedgehog mammarenavirus-es from	GenBank accession numbers	NP aa identity in % to					
		MEMV (OP191656)	ER8 (PQ441960)	ER15 (PQ441962)	ER27 (PQ441964)	ER33 (PQ441966)	ER39 (PQ441968)
Russia	PQ041966	84%	85%	86%	85%	86%	84%
	PQ041967	76%	76%	76%	77%	77%	76%
	PQ059274	85%	88%	88%	88%	88%	84%
	PQ059275	76%	77%	76%	77%	77%	75%
	PQ059279	84%	87%	87%	87%	87%	84%
Italy	PP934159	78%	79%	79%	79%	79%	78%
	PP934160	78%	79%	78%	79%	78%	77%
	PP934161	78%	79%	79%	79%	79%	78%
	PP934162	76%	79%	78%	78%	78%	76%
China	OP899821	75%	77%	77%	78%	77%	75%
	OP899822*	53%	53%	53%	53%	53%	53%

*Hedgehog mammarenavirus XIAMEN-10 (OP899822) from China belongs to the species *Mammarenavirus wenzhouense*.

## Discussion

In this study, additional novel mammarenavirus sequences were found in faecal samples from hedgehogs (*Erinaceus roumanicus*) collected from different sampling areas in Hungary (Fig. 1, Table S1). Based on these results, mammarenaviruses are present in Northern white-breasted hedgehogs in a wider geographical area in Hungary and, based on the unpublished mammarenavirus sequences, in other hedgehog species in Europe and Asia (Italy, Russia and China). These data confirm that hedgehogs, as novel hosts, are reservoirs of wide range of previously unknown mammarenavirus^14^.

The hierarchical clustering approach employed in this study effectively grouped the protein sequences into distinct clusters based on sequence distances, with groupings representing sequences from Hungary, Italy, Russia, and China suggesting potential host-virus or geographic region-virus relationships (Fig. 5). By using the factoextra package, hierarchical clustering classifies sequences based on amino acid identities, making it a valuable tool for visualizing and interpreting sequence distances. Unlike phylogenetic analysis, which uncovers evolutionary or temporal relationships, hierarchical clustering can also reveal non-evolutionary similarities, such as functional or structural patterns, which might not be apparent in phylogenetic trees. This method thus provides insights into previously unidentified relationships, contributing to a more precise separation of sequences and highlighting subtle distinctions that could lead to new functional hypotheses. High genetic diversity was found among the analyzed mammarenavirus sequences in hedgehogs from Hungary, Italy, Russia and China (Table 2). It can be observed that some mammarenavirus strains and lineages detected from hedgehogs living geographically closer to each other are genetically much more closely related to each other (Fig. 4). The Hungarian, Italian and Chinese mammarenaviruses detected from different hedgehog species (*Erinaceus roumanicus*, *Erinaceus europeus*, *Erinaceus amurensis*) are genetically distinct from each other. The hedgehog mammarenaviruses from Russia detected in undetermined hedgehog species. In Russia, four species of hedgehogs (*Erinaceus roumanicus*,* Erinaceus europaeus*,* Mesechinus dauuricus and Erinaceus amurensis*) occur and by the most common is *Erinaceus roumanicus* with a distribution from the westernmost parts of the country eastwards to the Ob river^22^. Based on the current ICTV species demarcation criteria, (which are: the virus shares less than 80% nucleotide sequence identity in the S-segment and less than 76% identity in the L-segment; association of the virus with a distinct main host or a group of sympatric hosts; dispersion of the virus in a distinct defined geographical area; association (or lack thereof) with human disease; and the virus shares less than 88% NP amino-acid sequence identity with other mammarenaviruses)^1^, there are potentially five novel hedgehog-related mammarenavirus species (Table 2, Table S2, Figs. 4 and 5), in addition to *Mammarenavirus mecsekense* and *Mammarenavirus wenzhouense*) in the genus *Mammarenavirus.* We tentatively named the group representing the Hungarian mammarenavirus strains ER8/2023/HUN, ER15/2023/HUN, ER27/2023/HUN and ER33/2023/HUN as Pannonia mammarenavirus (PANV) based on the name of the province (Pannonia) of the Roman Empire partly covered by the geographic study area. In consequence, *Erinaceus roumanicus* is a reservoir of minimum of two mammarenavirus species.

Interestingly, the intergenic region of the S-segment (S-IGR) is significantly longer in ER8/2023/HUN, ER15/2023/HUN, ER27/2023/HUN and ER33/2023/HUN (Table 1) than the S-IGR nt sequence length in other mammarenaviruses. Based on the sequence and secondary RNA structure analysis of these strains we found two, genetically highly similar stem-loop structures with conserved self-complementary nt motifs in this genome part (Figs. 2 and 3) instead of a usual single hairpin among mammarenaviruses^2,23^. These twin stem-loop structures with conserved nucleotide motifs were also present in three hedgehog mammarenaviruses from Russia (unpublished), the Mopeia^24^, Morogoro and Luna Bicuar Old World mammarenavirus S-IGRs (Fig. 2).

This study demonstrates that genetically diverse mammarenaviruses with unique genome motifs are present and circulate endemically in hedgehogs in geographically different regions in Europe. These mammarenaviruses potentially represent more than one taxonomically novel mammarenavirus species with currently unknown pathogenic potential.

## Methods

Faecal samples were collected from the Northern white-breasted hedgehog (*Erinaceus roumanicus*) from different geographic regions of Hungary between 2023 and 2024 (Table S1). Faecal specimens were collected from carcasses of road-hit hedgehogs or live animals (cared for in an official animal shelter and waiting for re-wild) by qualified biologists with valid permission (the National Inspectorate for Environment, Nature and Water: 4018-4/2015). Animal handling was done in accordance with relevant guidelines and regulations. In both carcasses and live animals, faeces samples of 2–5 grams were transferred with disposable pipettes into sterile Eppendorf tubes and DNA/RNAShield (Cambridge Bioscience) was added to prevent the degradation of nucleic acids. We isolated RNA from the samples upon arrival at the laboratory than were stored at -80°C. The host origin (*Erinaceus roumanicus*) of the mammarenavirus-positive specimens confirmed by PCR and Sanger sequencing method (PQ848113-PQ848117) with the in-house-designed cytochrome-b primers F, 5’-GAGGCGCTACAGTCATTACTA-3’; R, 5’-CATTGACTTACAGGTCGGAAT^14^. Viral RNA was isolated from stool samples using TRI Reagent (MRC, Cincinnati, OH, USA) based on the manufacturer’s instructions. The samples were screened by RT-semi-nested PCR using the primers (Arena-screen-F1 5’-TCWTATAAAGARCARGTYGG-3’, Arena-screen-F2 5’-AAATGGGGNCCNATGATGT-3’, Arena-screen-R 5’-ATYTGATCATCACTWGAWGT-3’) designed on the L-segment (RdRp) of Alxa virus (GenBank acc. No. KY432892), lymphocytic choriomeningitis virus (LCMV, AY847351), Lassa virus (MG812678) and Mecsek Mountains virus (MEMV, OP191655). The following primers were also designed for the RT-PCR detection of the S genome segment of ER8/2023/HUN and ER15/2023/HUN (ER8/ER15-F: 5’-GCACMGKGGATCCTAGGCTT-3’, ER8/ER15-R1: 5’-TCCATYAAACCCARTGGTGTT-3’, ER8/ER15-R2: 5’-AAATACCARAATTTAGTGTAGTTA-3’); ER27/2023/HUN and ER39/2023/HUN (ER27/ER39-F: 5’-CCTCATAGACTAACATCAATGG-3’, ER27/ER39-R: 5’-GTDGTHAGRGTTTGGGATGT-3’) and ER33/2023/HUN (ER33-F: 5’-GAGGTTGTTAATATTGTGATTATGGT-3’, ER33-R: 5’-CTTGGTGGAAATATGATAGA-3’), respectively, based on the S-segments of Alxa virus (KY432893), lymphocytic choriomeningitis viruses (LCMV, AY547350, AB627953), and Mecsek Mountains virus (MEMV, OP191656). For preparing L-segment cDNA, we used Arena-screen-R, RiboLock RNase Inhibitor (40 U/µL, ThermoFisher Scientific, Waltham, MA, USA) and M-MLV Reverse Transcriptase (200 U/µL, Promega) the reaction parameters were set based on the manufacturer’s instructions. For the first and second round PCR reactions, DreamTaq DNA Polymerase (5 U/µL, ThermoFisher Scientific) and/or Phusion High-Fidelity DNA Polymerase (2 U/µl, ThermoFisher Scientific) were used. The PCR protocol started with initial denaturation of 95 °C for 2 min, followed by 39 cycles of 95 °C for 30 s denaturation, 20 s of annealing and 75 °C of extension. After 39 cycles, a final step of 75 °C for 5 min was performed than cooled the samples to 4 °C. The annealing temperatures were applied based on the melting point of the primers. The extension time was determined based on the expected length of the PCR product and the DNA synthesis rate of the polymerase (~ 1 min/kilobase). In the first and second round PCR reactions we expected a ~ 632-638-bp long outer and a ~ 419-425-bp long inner amplicons, respectively. BioRad C1000 Touch™ Thermal Cycler (BioRad, Hercules, California, USA) was used for cDNA synthesis and PCR reactions. Positive and negative (nuclease-free water) controls were used in each reactions. The genomes of the novel mammarenaviruses were determined by “primer walking” method using primers (*N* = 88, excluding the additional control reaction primers designed to check the existing genome parts) designed on the L- and S-segments of the above listed MEMV, LCMV, Alxa viruses, and the study viruses. The primer sequences are available from the authors upon request. The terminal 19–20 nucleotide long sequences of the segments may match the primer sequences as indicated also in GenBank. PCR products were sequenced directly in both directions using an automated DNA sequencer (3500 Genetic Analyzer, Applied Biosystems, Japan). The methods of sequence- and phylogenetic analyses and the hierarchical clustering are attached to the text of the figures.

## Electronic supplementary material

Below is the link to the electronic supplementary material.

Supplementary Material 1

## Data Availability

Nucleotide sequence data reported have been deposited in the DDBJ/EMBL/GenBank databases under the accession numbers PQ441959-PQ441968 and PQ848113-PQ848117.Sequence data is also provided as “Related electronic material” to the manuscript.

## References

[1] Radoshitzky, S. R. et al. ICTV virus taxonomy profile: *Arenaviridae* 2023. *J. Gen. Virol.* **104**, 001891 (2023). 37698490 10.1099/jgv.0.001891 PMC10720992

[2] Iwasaki, M., Cubitt, B., Sullivan, B. M. & de la Torre, J. The high degree of sequence plasticity of the arenavirus noncoding intergenic region (IGR) enables the use of a nonviral universal synthetic IGR to attenuate arenaviruses. *J. Virol.*, **90** (6), 3187–3197 . 10.1128/JVI.03145-15 PMC4810653 26739049

[3] Burri, D. J. et al. Differential recognition of old world and new world arenavirus envelope protein glycoproteins by subtilisin kexin isozyme 1 (SKI-1/Site 1 protease (S1P). *J. Virol.* **87** (11), 6406–6414 (2013). 23536681 10.1128/JVI.00072-13 PMC3648084

[4] Katz, M. et al. Structure and receptor recognition by the Lassa virus spike complex. *Nature* **603**, 174–179 (2022). 35173332 10.1038/s41586-022-04429-2

[5] Hepojoki, J. et al. Characterization of Haartman Institute Snake virus-1 (HISV-1) and HISV-like viruses: Tthe representatives of genus Hartmanivirus, family *Arenaviridae*. *PLoS Pathog.* **14** (11), e1007415 (2018). 30427944 10.1371/journal.ppat.1007415 PMC6261641

[6] Shi, M. et al. The evolutionary history of vertebrate RNA viruses. *Nature* **556** (7700), 197–202 (2018). 29618816 10.1038/s41586-018-0012-7

[7] Chen, Y. M. et al. RNA viromes from terrestrial sites across China expand environmental viral diversity. *Nat. Microbiol.* **7** (8), 1312–1323 (2022). 35902778 10.1038/s41564-022-01180-2

[8] Luo, X. L. et al. Emergence of an ancient and pathogenic mammarenavirus. *Emerg. Microbes Infect.* **12** (1), e2192816 (2023). 36939609 10.1080/22221751.2023.2192816 PMC10337645

[9] Downs, W. G., Anderson, C. R., Spence, L., Aitken, T. H. G. & Greenhall, A. H. Tacaribe virus, a new agent isolated from Artibeus bats and mosquitoes in Trinidad, West Indies. *Am. J. Trop. Med. Hyg.* **12**, 640–646 (1963). 22324073 10.4269/ajtmh.1963.12.640

[10] Cui, X. et al. Virus diversity, wildlife-domestic animal circulation and potential zoonotic viruses of small mammals, pangolins and zoo animals. *Nat. Commun.* **14** (1), 2488 (2023). 37120646 10.1038/s41467-023-38202-4 PMC10148632

[11] Sayler, K. A. et al. Isolation of Tacaribe virus, a caribbean arenavirus, from host-seeking *Amblyomma americanum* ticks in Florida. *PloS One*, **9**(12), e115769 (2014). 10.1371/journal.pone.0115769 PMC4275251 25536075

[12] Li, K. et al. Isolation and characterization of a novel arenavirus harbored by rodents and shrews in Zhejiang Province, China. *Virology* **476**, 37–42 (2015). 25506671 10.1016/j.virol.2014.11.026

[13] Wu, Z. et al. Detection of hantaviruses and arenaviruses in three-toed jerboas from the Inner Mongolia Autonomous Region, China. *Emerg. Microbes Infect.* **7**, 35 (2018). 29559618 10.1038/s41426-018-0036-y PMC5861045

[14] Reuter, G., Boros, Á., Takáts, K., Mátics, R. & Pankovics, P. A novel mammarenavirus (family *Arenaviridae*) in hedgehogs (*Erinaceus roumanicus*) in Europe. *Arch. Virol.* **168** (7), 174 (2023). 37291370 10.1007/s00705-023-05804-8 PMC10250479

[15] Brisse, M. E. & Ly, H. Hemorrhagic fever-causing arenaviruses: Lethal pathogenesis and potent immune suppressors. *Front. Immunol.* **10**, 372 (2019). 30918506 10.3389/fimmu.2019.00372 PMC6424867

[16] Vilibic-Cavlek, T. et al. Lymphocytic choriomeningitis - emerging trends of a neglected virus: A narrative review. *Trop. Med. Infect. Dis.* **6** (2), 88 (2021). 34070581 10.3390/tropicalmed6020088 PMC8163193

[17] Kuhn, J. H. et al. taxonomic update of RNA-directed RNA polymerase-encoding negative-sense RNA viruses (realm *Riboviria*: kingdom *Orthornavirae*: phylum *Negarnaviricota*). *J .Gen. Virol.* (2025) in press (2024). 10.1099/jgv.0.001864 PMC10721048 37622664

[18] Morin, B. et al. The N-terminal domain of the arenavirus L protein is an RNA endonuclease essential in mRNA transcription. *PLoS Pathog*, **6**(9), e1001038 (2010). 10.1371/journal.ppat.1001038 PMC2940758 20862324

[19] Fehling, S. K., Lennartz, F. & Strecker, T. Multifunctional nature of the arenavirus RING finger protein Z. *Viruses* **4**, 2973–3011 (2012). 23202512 10.3390/v4112973 PMC3509680

[20] Wang, J., Danzy, S., Kumar, N., Ly, H. & Liang, Y. Biological roles and functional mechanisms of arenavirus Z protein in viral replication. *J. Virol.* **86** (18), 9794–9801 (2012). 22761375 10.1128/JVI.00385-12 PMC3446593

[21] Pasquato, A., Cendron, L. & Kunz, S. Cleavage of the glycoprotein of arenaviruses. In: (eds. Böttcher-Friebertshäuser, E., Garten, W. & Klenk, H.) Activation of Viruses by Host Proteases (Springer, Cham. (2018).

[22] Amori, G. et al. Erinaceus roumanicus (amended version of 2016 assessment). *IUCN Red List. Threatened Species*. e.T136344A197508156 10.2305/IUCN.UK.2021-1.RLTS.T136344A197508156.en (2021).

[23] Kiening, M., Weber, F. & Frishman, D. Conserved RNA structures in the intergenic regions of ambisense viruses. *Sci. Rep.* **7**, 16625 (2017). 29192224 10.1038/s41598-017-16875-4 PMC5709424

[24] Wilson, S. M. & Clegg, J. C. Sequence analysis of the S RNA of the African arenavirus Mopeia: An unusual secondary structure feature in the intergenic region. *Virology* **180** (2), 543–552 (1991). 1989384 10.1016/0042-6822(91)90068-m

[25] Löytynoja, A. & Goldman, N. webPRANK: A phylogeny-aware multiple sequence aligner with interactive alignment browser. *BMC Bioinform.* **11** (1), 1–7 (2010). 10.1186/1471-2105-11-579 PMC3009689 21110866

[26] Radoshitzky, S. R. et al. ICTV virus taxonomy profile: *Arenaviridae*. *J. Gen. Virol.* **100** (6), 1200–1201 (2019). 31192784 10.1099/jgv.0.001280 PMC12139605

[27] Trifinopoulos, J., Nguyen, L. T., von Haeseler, A. & Minh, B. Q. W-IQ-TREE: A fast online phylogenetic tool for maximum likelihood analysis. *Nucleic Acids Res.* **44** (W1), W232–W235 (2016). 27084950 10.1093/nar/gkw256 PMC4987875

[28] Kumar, S., Stecher, G., Li, M., Knyaz, C. & Tamura, K. MEGA X: Molecular evolutionary genetics analysis across computing platforms. *Mol. Biol. Evol.* **35** (6), 1547 (2018). 29722887 10.1093/molbev/msy096 PMC5967553

[29] Borg, I. & Groenen, P. J. F. *Modern Multidimensional Scaling: Theory and Applications* (Springer, 2005).

[30] Jolliffe, I. T. & Cadima, J. Principal component analysis: A review and recent developments. *Philos. Trans. A Math. Phys. Eng. Sci.* **374** (2065), 20150202 (2016). 10.1098/rsta.2015.0202 PMC4792409 26953178

