# Supplementary material for: Novel relatives of Mecsek Mountains mammarenavirus (family Arenaviridae) in hedgehogs living in different sampling areas in Hungary
Source: Sci Rep. 2025 Jan 23;15:2907. doi: 10.1038/s41598-025-87108-2 (PMC11758386; doi:10.1038/s41598-025-87108-2)
Supplement: Supplementary file 1 — Supplementary Material 1 [file 41598_2025_87108_MOESM1_ESM.docx]

**Supplementary information**

**Table S1.** Summary of the sampled specimens from hedgehogs (Erinaceus roumanicus) and the RT-PCR results for mammarenavirus. †: samples from carcasses of road-hit hedgehogs; #: samples from live hedgehogs cared for in an official animal shelter

| Sample ID | Sample type | Sampling time | Geographical location | RT-PCR screening results |
| --- | --- | --- | --- | --- |
| ER1 | Stool# | Jan 15, 2023 | Csór  47°12′14″N 18°15′25″E | negative |
| ER2 | Stool# | Jan 15, 2023 | Szigetvár  46°02′51″N 17°47′58″E | negative |
| ER3 | Stool# | Jan 17, 2023 | Pákozd  47°13′16″N 18°32′42″E | negative |
| ER4 | Stool# | Apr 1, 2023 | Simonfa  46°17′01″N 17°49′23″E | negative |
| ER5 | Stool# | Apr 1, 2023 | Simonfa  46°17′01″N 17°49′23″E | negative |
| ER6 | Stool† | May 21, 2023 | Érd  47°23′N.18°55′E | negative |
| ER7 | Stool# | June 6, 2023 | Veresegyháza  47°39′25″N 19°17′05″E | negative |
| ER8 | Stool# | June 8, 2023 | Úrhida  47°07′50″N 18°19′55″E | **positive** |
| ER9 | Stool# | June 21, 2023 | Csór  47°12′14″N 18°15′25″E | negative |
| ER10 | Stool† | June 21, 2023 | Székesfehérvár  47°11′20″N 18°24′50″E | negative |
| ER11 | Stool# | June 25, 2023 | 16th district of Budapest  47°31′13″N 19°10′26″E | negative |
| ER12 | Stool# | June 25, 2023 | Székesfehérvár  47°11′20″N 18°24′50″E | negative |
| ER13 | Stool# | June 26, 2023 | 4th district of Budapest  47° 34′N 19°05′E | negative |
| ER14 | Stool# | June 27, 2023 | Simonfa  46°17′01″N 17°49′23″E | negative |
| ER15 | Stool# | June 27, 2023 | Székesfehérvár  47°11′20″N 18°24′50″E | **positive** |
| ER16 | Stool# | June 28, 2023 | Székesfehérvár  47°11′20″N 18°24′50″E | negative |
| ER17 | Stool# | July 1, 2023 | Gellért Hill Budapest  47°29′13″N 19°02′50″E | negative |
| ER18 | Stool# | July 1, 2023 | 3rd district of Budapest  47°34′N 19°02′E | negative |
| ER19 | Stool# | July 1, 2023 | 4th district of Budapest  47° 34′N 19°05′E | negative |
| ER20 | Stool# | July 1, 2023 | Székesfehérvár  47°11′20″N 18°24′50″E | negative |
| ER21 | Stool# | July 2, 2023 | 16th district of Budapest  47°31′13″N 19°10′26″E | negative |
| ER22 | Stool# | July 3, 2023 | 12th district of Budapest  47°30′N. 19° 00′E | negative |
| ER23 | Stool# | July 28, 2023 | Pomáz  47°38′51″N 19°01′37″E | negative |
| ER24 | Stool# | Aug 8, 2023 | 21st district of Budapest  47°25′N 19°05′E | negative |
| ER25 | Stool# | Aug 9, 2023 | 16th district of Budapest  47°31′13″N 19°10′26″E | negative |
| ER26 | Stool# | Aug 9, 2023 | 17th district of Budapest  47°28′49″N 19°16'00″E | negative |
| ER27 | Stool# | Aug 13, 2023 | Budapest  47°29′54″N 19°02′27″E | **positive** |
| ER28 | Stool# | Aug 15, 2023 | Budapest 47°29′54″N19°02′27″E | negative |
| ER29 | Stool# | Aug 16, 2023 | 3rd district of Budapest  47°34′N 19°02′E | negative |
| ER30 | Stool# | Aug 19, 2023 | 4th district of Budapest  47° 34′N 19°05′E | negative |
| ER31 | Stool# | Aug 30, 2023 | 16th district of Budapest  47°31′13″N 19°10′26″E | negative |
| ER32 | Stool# | Aug 31, 2023 | 14th district of Budapest  47°30′45″N 19°06′30″E | negative |
| ER33 | Stool# | Aug 31, 2023 | Fót  47°36′33″N 19°11′34″E | **positive** |
| ER34 | Stool# | Aug 31, 2023 | 15th district of Budapest  47°28′N 19°05′E | negative |
| ER35 | Stool# | Sept 3, 2023 | 11th district of Budapest 47°28′30″N 19°02′24″E | negative |
| ER36 | Stool# | Sept 4, 2023 | Gyál  47°22′56″N 19°12′49″E | negative |
| ER37 | Stool# | Sept 5, 2023 | 18th district of Budapest  47°26′38″N 19°10′35″E | negative |
| ER38 | Stool# | Sept 8, 2023 | 2nd district of Budapest  47°31′N 19°01′E | negative |
| ER39 | Stool# | Sept 15, 2023 | Szár  47°28′39″N 18°30′58″E | **positive** |
| ER40 | Stool# | Sept 27, 2023 | 20th district of Budapest  47°26′06″N 19°07′00″E | negative |
| ER41 | Stool# | Oct 2, 2023 | Fót  47°36′33″N 19°11′34″E | negative |
| ER42 | Stool# | Oct 7, 2023 | Szigetcsép  47°16′N 18°59′E | negative |
| ER43 | Stool# | Oct 15, 2023 | Taksony  47°19′55″N 19°03′47″E | negative |
| ER44 | Stool# | Oct 19, 2023 | Halásztelek  47°21′39″N 18°59′16″E | negative |
| ER45 | Stool# | Oct 23, 2023 | 16th district of Budapest  47°31′13″N 19°10′26″E | negative |
| ER46 | Stool# | Oct 24, 2023 | Budapest  47°29′54″N 19°02′27″E | negative |
| ER47 | Stool# | Nov 11, 2023 | 16th district of Budapest  47°31′13″N 19°10′26″E | negative |
| ER48 | Stool# | Nov 13, 2023 | Dunaharaszti  47°21′19″N 19°05′04″E | negative |
| ER49 | Stool# | Nov 22, 2023 | Biatorbágy  47°28′27″N 18°49′25″E | negative |
| ER50 | Stool# | Dec 21, 2023 | 15th district of Budapest  47°33′32″N 19°07′10″E | negative |
| ER51 | Stool# | Jan 4, 2024 | Budapest  47°29′54″N19°02′27″E | negative |
| ER52 | Stool# | Jan 7, 2024 | 4th district of Budapest  47° 34′N 19°05′E | negative |
| ER53 | Stool# | Jan 22, 2024 | Biatorbágy  47°28′27″N 18°49′25″E | negative |
| ER54 | Stool# | Jan 22, 2024 | Szigetszentmiklós  47°20′43″N 19°02′54″E | negative |
| ER55 | Stool# | Jan 22, 2024 | Budapest  47°29′54″N 19°02′27″E | negative |
| ER56 | Stool# | Jan 24, 2024 | 16th district of Budapest  47°31′13″N 19°10′26″E | negative |
| ER57 | Stool# | Jan 27, 2024 | 12th district of Budapest  47°30′N. 19° 00′E | negative |
| ER58 | Stool# | Feb 2, 2024 | 12th district of Budapest  47°30′N 19° 00′E | negative |
| ER59 | Stool# | Feb 8, 2024 | Budapest  47°29′54″N 19°02′27″E | negative |

**Table S2.**

| Hedgehog mammarenavirus L-segment nucleotide sequence(s) from | GenBank accession numbers | nt identity in % to | | | | | | | | |
| --- | --- | --- | --- | --- | --- | --- | --- | --- | --- | --- |
|  |  | Russia | | | | Italy | | | | China |
|  |  | PQ059273 | PQ059276 | PQ059277 | PQ059278 | PP934155 | PP934156 | PP934157 | PP934158 | OP899820 (partial) |
| Russia | PQ059273 | - | - | - | - | - | - | - | - | - |
|  | PQ059276 | 69% | - | - | - | - | - | - | - | - |
|  | PQ059277 | 82% | 69% | - | - | - | - | - | - | - |
|  | PQ059278 | 99% | 69% | 82% | - | - | - | - | - | - |
| Italy | PP934155 | 66% | 66% | 67% | 66% | - | - | - | - | - |
|  | PP934156 | 66% | 65% | 67% | 66% | 90% | - | - | - | - |
|  | PP934157 | 66% | 65% | 66% | 66% | 89% | 87% | - | - | - |
|  | PP934158 | 66% | 66% | 66% | 66% | 89% | 88% | 93% | - | - |
| China | OP899820 (partial) | 60% | 59% | 60% | 60% | 60% | 60% | 60% | 60% | - |

| Hedgehog mammarenavirus S-segment nucleotide sequence(s) from | GenBank accession numbers | nt identity in % to | | | | | | | | | |
| --- | --- | --- | --- | --- | --- | --- | --- | --- | --- | --- | --- |
|  |  | Russia | | | | | Italy | | | | China |
|  |  | PQ041966 | PQ041967 (partial) | PQ059274 | PQ059275 (partial) | PQ059279 | PP934159 | PP934160 | PP934161 | PP934162 | OP899821 |
| Russia | PQ041966 | - | - | - | - | - | - | - | - | - | - |
|  | PQ041967 (partial) | 63% | - | - | - | - | - | - | - | - | - |
|  | PQ059274 | 88% | 64% | - | - | - | - | - | - | - | - |
|  | PQ059275 (partial) | 65% | 88% | 65% | - | - | - | - | - | - | - |
|  | PQ059279 | 86% | 61% | 95% | 62% | - | - | - | - | - | - |
| Italy | PP934159 | 70% | 66% | 69% | 67% | 66% | - | - | - | - | - |
|  | PP934160 | 69% | 65% | 69% | 67% | 67% | 93% | - | - | - | - |
|  | PP934161 | 70% | 65% | 69% | 67% | 67% | 92% | 91% | - | - | - |
|  | PP934162 | 70% | 66% | 69% | 67% | 66% | 81% | 81% | 82% | - | - |
| China | OP899821 | 64% | 63% | 65% | 66% | 63% | 67% | 67% | 67% | 66% | - |
